# Quadruplex-and-Mg^2+^ Connection (QMC) of DNA

**DOI:** 10.1038/srep12996

**Published:** 2015-08-12

**Authors:** Besik Kankia

**Affiliations:** 1 Department of Chemistry and Biochemistry, The Ohio State University, Columbus OH 43210, USA

## Abstract

This work highlights a novel method of coupling of nucleic acids through formation of an extraordinary stable, specific and fully reversible quadruplex-and-Mg^2+^ connection (QMC). QMC employs the monomolecular tetrahelical architecture of DNA and has two key components: (i) shape complementarity between QMC partners, which is introduced by specific modifications of the quadruplexes, and (ii) Mg^2+^ ions. The on-rate of QMC formation is between 10^5^–10^6^ M^−1^ s^−1^, while the off-rate is undetectable even at 80 °C. However, QMC dissociates rapidly upon removal of Mg^2+^ ions (i.e., by EDTA). QMC is characterized by high specificity, as even a single-nucleotide modification of one of the connectors inhibits complex-formation. QMC has the potential to revolutionize biotechnology by introducing a new class of capture molecules with major advantages over traditional systems such as streptavidin-biotin. The advantages include reversibility, multiplexing, higher stability and specificity, longer shelf life and low cost.

Recently, we described a programmable single-molecular tetrahelical architecture of DNA that employs G (guanine)-quartets as a structural element^1^. The monomeric unit of the architecture, G_3_TG_3_TG_3_TG_3_ (G3T), is folded in an intramolecular quadruplex with three G-quartets. In the tetrahelical architecture, the G3T monomers are stacked on each other forming an extraordinarily stable uninterrupted polymer. For instance, a (G3T)_3_ trimer consists of nine G-quartets (Fig. 1a,b). This assembly is so stable that even the dimer, (G3T)_2_, melts above 100 °C at very low ionic strength (i.e., 1 mM KCl)^1^.

While K^+^ is an efficient quadruplex-forming cation, Mg^2+^ ions do not facilitate quadruplex formation^2^. However, in this work we show that Mg^2+^ ions are able to restore the tetrahelical structure of (G3T)_2_ disrupted by specific modifications (i.e., insertion or deletion of nucleotides) (Fig. S1). Based on these results, we proposed stable and fully reversible quadruplex-and-Mg^2+^ connection (QMC) of DNA molecules. The main assumption of QMC is that, in the presence of Mg^2+^ ions, two specifically designed quadruplexes will stack on each other to form a stable uninterrupted tetrahelix. To maintain the folding pattern of the single-molecular tetrahelix (Fig. 1b) and to make the interaction specific, we introduced shape complementarity, i.e., an interlocking mechanism, by nicking (G3T)_3_ within the middle (2^nd^) G3T unit (Fig. 1). For instance, nicking between G23 and G24 creates a pair of QMC connectors; the left connector (LC23) consists of two G3T monomers, one full-length and another missing the last guanine. The right connector (RC24) represents a G3T unit with one extra guanine at the 5′-end (Fig. 1a). Despite the modifications, each separate connector is capable of quadruplex formation. Upon mixing they interact with each other and fold into the uninterrupted tetrahelix with one interface G-quartet (i.e., G-quartet formed by guanines from both connectors: three from LC23 and one from RC24 (dark G-quartet in Fig. 1c)). Thus the interface quadruplex serves as an interlocking mechanism, similarly to a jigsaw puzzle, and secures proper folding of the product tetrahelix (Fig. 1c). Similarly, LC13 and RC1 4 (Fig. 1a) represent another QMC pair nicked between G13 and G14 with another interface G-quartet (Fig. 1d). Note the mechanism of QMC, described above, is explained in terms of (G3T)_3_ nicking. Alternatively, QMC connectors can be considered as (G3T)_2_ and G3T with the 3′-end nucleotides of the former transferred to the 5′-end of the latter.

## Results and Discussion

### Thermal unfolding experiments of 2AP-containing QMC partners

We performed a series of fluorescence unfolding experiments using three different systems: LC13/RC14, LC23/RC24 and LC24/RC25. While the first two systems possess specific interlocking mechanisms, the latter represents a pair of blunt-ended quadruplexes with both ends terminated by perfect G-quartet without extra or missing Gs, (G3T)_2_ and G3T. In these experiments, one of the partners from each pair was labeled with 2-aminopurine (2AP) (i.e., LC13, RC24 and RC25) and its thermal stability, *T*_m_, was determined in the presence of the matching and mismatching (cross-binding) partners with and without Mg^2+^ ions. For instance, the *T*_m_ of 2AP-LC13 was determined in the presence of matching RC14 and cross-binding LC23 (Fig. S2). The results revealed that (i) in the absence of Mg^2+^ ions, none of the pairs, including matching connectors, demonstrate any significant stabilization; (ii) the blunt-ended pair, or the system without any locking mechanism, (LC24/RC25) did not show stabilization even in the presence of Mg^2+^; (iii) none of the mismatched or cross-binding partners, even in the presence of Mg^2+^, showed stabilization; and (iv) only matching pairs with the interlocking mechanism demonstrate a large increase in *T*_m_ in the presence of Mg^2+^ (Fig. S2), which are attribute to QMC formation. Thus, we conclude that (i) shape complementarity and Mg^2+^ ions are essential for QMC formation, and (ii) QMC is characterized by high specificity (even a single-nucleotide modification of the connectors restricts the process).

### Conditions for QMC formation

Since the length of QMC is <3 nm (0.33 nm rise per G-quartet^3^,^4^), it represents a convenient molecule to be studied by a fluorophore-quencher pair. The 5′-end of LC23 was tagged with carboxyfluorescein (FAM) and the 3′-end of RC24 was tagged with a black hole quencher (BHQ) and a systematic study has been performed to be sure that the quenching corresponds to QMC formation rather than some non-specific aggregation. Figure S3 demonstrates isothermal titration of FAM-LC23 by BHQ-RC24 under experimental conditions free from any non-specific aggregation discussed below, 250 nM FAM-LC23in 5 mM K^+^ and 2 mM Mg^2+^ at 50 °C. As expected, the titration revealed tight binding process with 1:1 stoichiometry.

Figure 2 demonstrates influence of BHQ-RC24 and Mg^2+^ on the fluorescence of FAM-LC23 at three different temperatures. Each panel contains two experiments. Adding Mg^2+^ ions followed by BHQ-RC24 (left) and adding BHQ-RC24 followed by Mg^2+^ (right). Adding BHQ-RC24 in the absence of Mg^2+^ ions (right) does not affect the fluorescence at any temperature. However, adding Mg^2+^ ions in the mixture is accompanied by a rapid fluorescence quenching, which well agrees with thermal unfolding experiments (Fig. S2) and indicates on the essential role of the divalent cations in QMC formation. Adding Mg^2+^ ions in the absence of BHQ-RC24 (left) also induced quenching effect, which must be due to self- aggregation or dimerization of FAM-LC23. This non-specific effect disappears at higher temperatures (Fig. 2) and/or lower reagent concentrations (Fig. S4). Therefore, experimental conditions in all tests were carefully selected to avoid any non-specific effects.

We also tested if QMC can be formed by K^+^ ions alone. Initially we monitored fluorescence of FAM-LC23 in the absence of BHQ-RC24 at varying concentration of K^+^ ions. Unlike Mg^2+^ ion, K^+^ ion did not induce self-aggregation of FAM-LC23 even at 1 M concentration (Fig. S5a). Next, BHQ-RC24 have been added into of FAM-LC23 dissolved in 0.1 M and 1 M K^+^, which revealed only partial quenching effects (Fig. S5b). Thus, under physiological conditions, QMC requires Mg^2+^ ions.

As shown in the previous section, LC13/RC14 and LC23/RC24 systems are characterized by stabilization effect (corresponding to strong interaction between them), while cross-binding experiments between them did not reveal any interaction. Thus, QMC has high specificity and has potential to be used in multiplexing experiments (i.e., detecting more than one target simultaneously). To further test specificity of QMC connectors, we performed additional studies. Fluorescence quenching experiments of FAM-LC23/BHQ-RC24 in the presence of the cross-binding (mismatched) partner, LC13, was conducted (Fig. S6). The experiments revealed, that LC13 is not able to restrict the quenching process, while “cold” analog of of FAM-LC23, LC23, efficiently interferes the quenching. This experiment clearly indicates that the cross-binding partners do not create a stable complex capable of restricting the interaction between matching QMC partners.

### QMC kinetics

**Figure 1:**
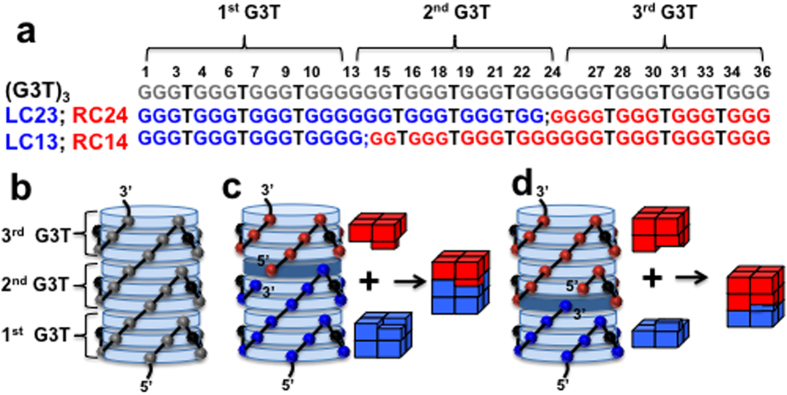
Design of QMC. (**a**) Nucleotide sequences of the QMC partners based on (G3T)_3_. (**b**) Three-dimensional model of the tetrahelical structure made of a single (G3T)_3_ strand. G-quartets are viewed as light blue disks. The grey spheres represent sugars of guanines. The black spheres, here and in panels (**c,d)**, represent sugars of unstructured thymidines forming the chain-reversal loops (thymines are omitted for clarity). (**c**) Model of QMC formed by LC23 and RC24 with one interface G-quartet (dark blue disc). The blue and red spheres represent sugars of guanines of LC23 and RC24, respectively. (Right) Schematic of the coupling process. (**d**) Model and schematic of QMC formed by LC13 and RC14.

**Figure 2:**
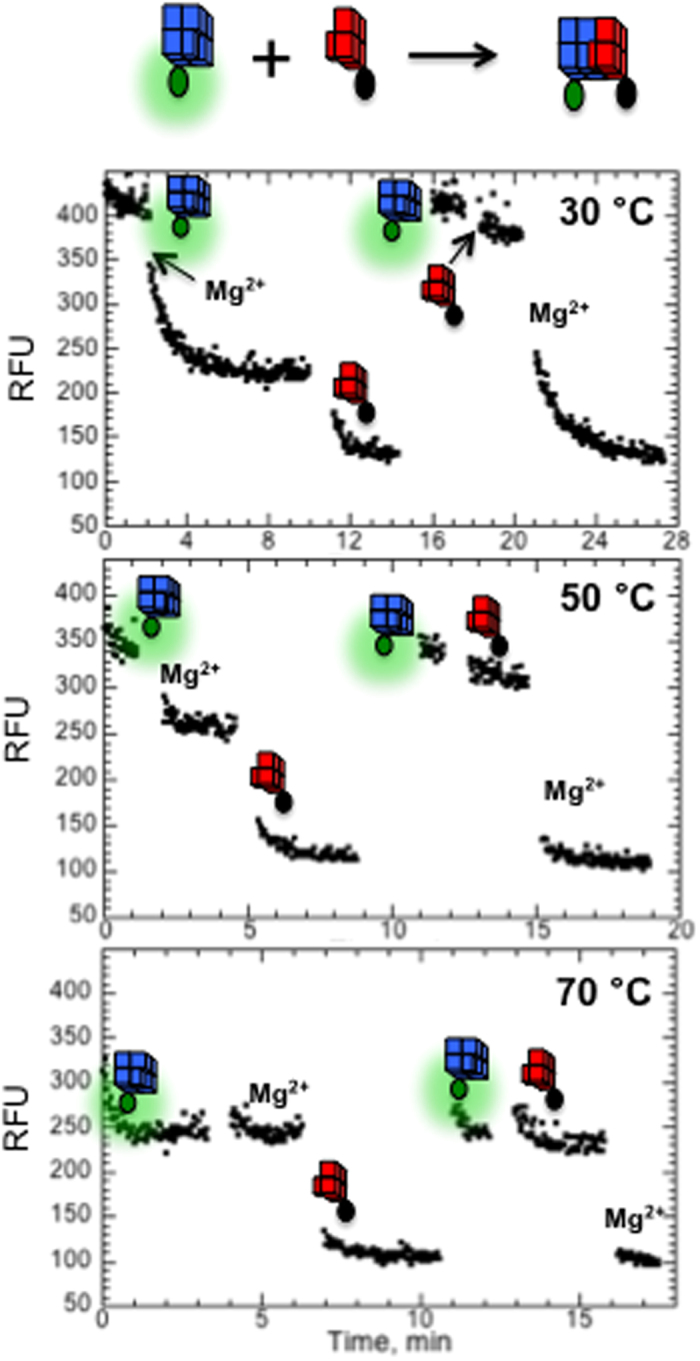
Fluorescence effects of 1 μM FAM-LC23 upon adding 10 mM MgCl_2_ and 1.25 μM BHQ-RC24 at different temperatures in 5 mM KCl.

On and off rates of QMC formation has been studied using the fluorophore-quencher system described in the previous section. The on-rate of the QMC formation increases steadily from 1.20 ± 0.04 × 10^5^ M^−1^ s^−1^ at 37 °C to 1.0 ± 0.1 × 10^6^ M^−1^ s^−1^ at 80 °C (Fig. S7). The rates are not as high as a diffusion-controlled binding mechanism would predict, 10^8^–10^9^ M^−1^ s^−1^ 5. This is consistent with the coupling mechanism shown in Fig. 1, which would suggest that the surface area of the binding interface represents ~15–25% of the total exposed area for each QMC partner. Therefore, not every collision between the quadruplexes is expected to result in the coupling. The on-rates are in general agreement with other high affinity biological bindings (i.e., nucleic acid hybridization^6^,^7^,^8^,^9^, or Streptavidin-Biotin interaction^10^,^11^). The temperature dependence of *k*_on_ reveals a small activation barrier of ~10 kcal mol^−1^, which could be attributed to the minor structural rearrangement upon forming the interface G-quartet.

**Figure 3:**
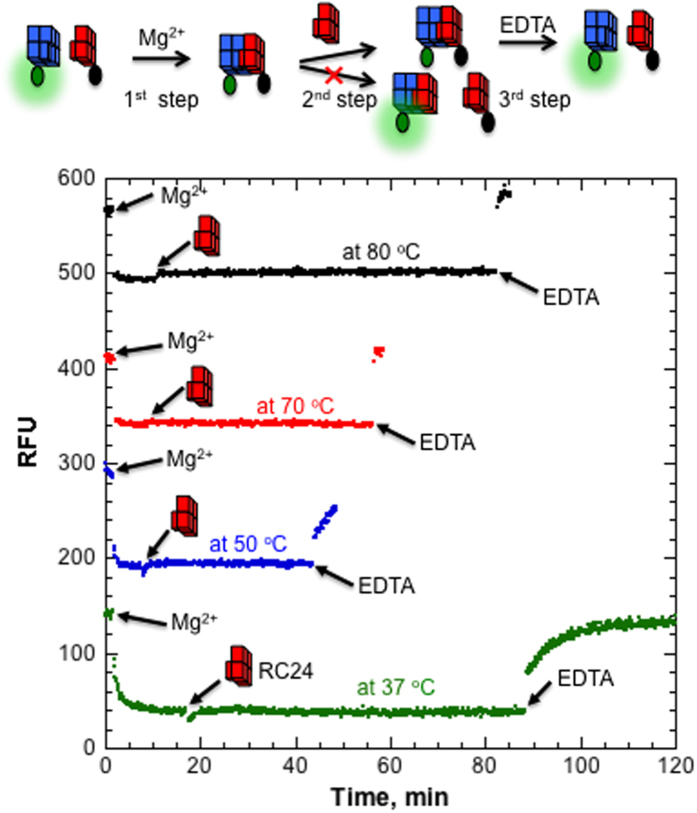
Kinetics of QMC formed by FAM-LC23/BHQ-RC24 measured at different temperatures in 5 mM KCl and 10 mM Tris-HCl. Each curve demonstrates fluorescence effects induced by sequential addition of 10 mM MgCl_2_, 16 μM RC24 and 25 mM EDTA. While Mg^2+^ and EDTA induce similar levels of fluorescence quenching and fluorescence recovery, respectively, 100-fold excess RC24 does not induce any fluorescence increase. For clarity, the curves at 50 °C, 70 °C and 80 °C are offset by 150, 300 and 450 RFU, respectively.

We could not detect any fluorescence recovery upon adding 100-fold excess RC24 into the preformed QMC (FAM-LC23/BHQ-RC24) even at 80 °C. To exclude any experimental artifacts, we performed additional control experiments. First, we tested validity of the RC24 oligonucleotide. BHQ-RC24 was added into the preformed FAM-LC23/RC24 complex. However, this did not reveal any quenching effect (Fig. S6), which clearly indicates that RC24 efficiently binds and occupies the binding site of FAM-LC23. In addition, we performed the following three-step experiment (Fig. 3). Initially, Mg^2+^ ions were added into the QMC mixture (FAM-LC23/BHQ-RC24) and fluorescence quenching was detected upon complex formation. After the minimum fluorescence signal was achieved, 100-fold excess RC24 was added, which did not reveal any measurable changes. We next added an excess of concentrated EDTA solution, which was accompanied by rapid fluorescence recovery due to QMC complex dissociation. Thus, QMC is a very stable complex, which does not dissociate to any measurable extent even at 80 °C, but rapidly dissociates upon chelating Mg^2+^ ions.

### QMC at solid support

**Figure 4:**
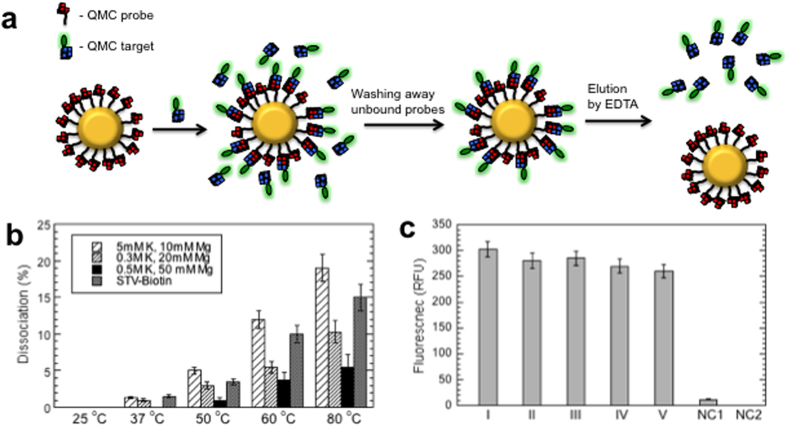
Characterization of QMC-covered magnetic beads. (**a**) Schematics of the QMC-capture mechanism and the binding capacity assay. (**b**) Thermal stability of QMC-beads estimated at different salt concentrations and STV-beads in 1 M NaCl, 0.5 mM EDTA. The beads were incubated for 5 min at the indicated temperatures, then supernatant was removed on a magnet and the fluorescence measured. (**c**) Multiple binding and elution of QMC-beads. Five consecutive immobilization and EDTA elution in 0.5 M KCl and 50 mM MgCl_2_ were performed demonstrating almost no effect in binding capacity. NC1 and NC2 correspond to negative controls, FAM labeled random oligonucleotide instead of QMC-target and MyOne carboxylic acid beads instead of QMC-beads, respectively.

To demonstrate QMC formation at solid support, we prepared QMC-functionalized magnetic beads by coating MyOne carboxylic acid (Invitrogen) beads with amino modified RC24 or QMC probe. After immobilization of QMC target, FAM-LC23, to the QMC-beads and washing away unbound targets, binding capacity and thermal stability of the beads have been estimated (Fig. 4a).

QMC-beads were prepared in the presence of four different amounts of the probe: 0.5, 1, 2 and 5 nmol probes per 1 mg MyOne beads. While all QMC-beads revealed significant amount of captured targets, the highest binding capacity was observed for the beads prepared in the presence of 1 nmol probe: 5 × 10^12^ target molecules per cm^2^, or one captured QMC target per ~20 nm^2^ of the bead surface area. Thus, average distance between QMC targets is 4.5 nm. Keeping in mind that DNA G-quartet stacks are highly charged molecules with a diameter of ~3 nm, binding capacity, reported here, might correspond to maximum density of QMC probes. The reported data well agrees with previous study on 18mer DNA oligomers captured by hybridization to its complementary strand immobimized at gold supports^12^. In this work the maximum target binding capacity was observed for the probes with average distance of 4 nm between them.

Control experiments performed on commercially available MyOne streptavidin beads (STV-bead), using FAM-T_20_-biotin as a target, revealed significantly higher binding capacity, corresponding to one STV-biotin binding per ~6 nm^2^. This value is in very good agreement with the manufactures suggested binding capacity and is determined by tetravalent nature of STV.

Figure 4B compares thermal stability of QMC and STV-biotin complexes immobilized at magnetic beads. As expected, with increase of ionic strength stability of QMC increases. This is due to reducing electrostatic interaction penalties between QMC partners at higher ionic strength. Figure 4C presents the results of multiple binding and elution of QMC targets from the same QMC-coated magnetic beads, which demonstrate no significant changes in binding capacity indicating on reusability of QMC-beads.

## Conclusion

We have outlined a new way to noncovalently couple DNA, QMC, which is based on single-molecular tetrahelical architecture of DNA. It will create new opportunities in many current applications in biotechnology (i.e., imaging, DNA amplification, sequencing). These applications depend on a streptavidin-biotin system for the capture of target molecules. However, streptavidin-biotin binding has several limitations. First, it is not readily reversible. Harsh conditions are required to break the bond, which usually denature both streptavidin and target proteins. Second, streptavidin denaturation is irreversible, which restricts the reuse of streptavidin-coated solid supports and complicates the coating process. Third, because biotin is a biological molecule, endogenous biotin can cause background and specificity problems in biotin-rich tissues and extracts. The same is true for samples containing endogenous biotin-binding proteins. Fourth, the streptavidin-biotin system is incapable of multiplexing and its shelf life is limited. All these problems can be addressed by QMC, which (i) is made of one of the most stable biopolymers with a long shelf life; (ii) forms an artificial tertiary structure not found in cells; (iii) is completely reversible; (iv) can be decoupled simply by the removal of Mg^2+^ ions, and (v) has a potential of multiplexing.

## Methods

### DNA oligonucleotides

DNA oligonucleotides were obtained from Integrated DNA Technologies. The concentration of DNA oligonucleotides was determined by measuring UV absorption at 260 nm. Oligonucleotide names and sequences from 5′ to 3′:

**G3T** or **RC25**: GGGTGGGTGGGTGGG

**AP-G3T** or **2AP-RC25**: GGG(2AP)GGGTGGGTGGG

**(G3T)**_**2**_ or **LC24**: GGGTGGGTGGGTGGGGGGTGGGTGGGTGGG

**G3T-T-G3T**: GGGTGGGTGGGTGGGTGGGTGGGTGGGTGGG

**(G3T)**_**3**_: GGGTGGGTGGGTGGGGGGTGGGTGGGTGGGGGGTGGGTGGGTGGG

**LC13**: GGGTGGGTGGGTGGGG

**AP-LC13:** GGG(2AP)GGGTGGGTGGGG

**RC14**: GGTGGGTGGGTGGGGGGTGGGTGGGTGGG

**LC23**: GGGTGGGTGGGTGGGGGGTGGGTGGGTGG

**RC24**: GGGGTGGGTGGGTGGG

**AP-RC24**: GGGG(2AP)GGGTGGGTGGG

**FAM-LC23**: FAM-TGGGTGGGTGGGTGGGGGGTGGGTGGGTGG

**BHQ-RC24**: GGGGTGGGTGGGTGGGT-BHQ

### UV and fluorescence unfolding

To determine melting temperatures, *T*_m_, UV absorption was recorded at or 295 nm as a function of temperature using a Varian UV–visible spectrophotometer (Cary 100 Bio). Fluorescence measurements of 2-aminopurine (2AP) (ex 310 nm, em 370 nm) were performed using a Varian spectrophotometer (Cary Eclipse). In a typical experiment, oligonucleotide samples were mixed and diluted into the desired buffers in optical cuvettes. The solutions were incubated at 95 °C for a few minutes in the cell holder prior to ramping to the desired starting temperature. The melting experiments were performed at a heating rate of 1 °C/min.

### Kinetic measurements

Kinetics was monitored through fluorescence effects of a fluorophore-quencher pair (carboxyfluorescein (FAM) and black hole quencher (BHQ)). The fluorescence was measured using a Varian spectrophotometer (Cary Eclipse) with 494 nm excitation and 520 nm emission.

In the on-rate assay, binding of FAM-LC23 to BHQ-RC24 results in fluorescence quenching. The binding was initiated by mixing equal volumes of 10 nM FAM-LC23 and 100 nM BHQ-RC24 solutions using a SFA20 (Hi-Tech) stopped-flow apparatus. The fluorescence was measured every 2.25 s. Both the optical cell and the solutions in the stopped-flow apparatus were equilibrated at the desired temperature. The reaction was followed until a constant fluorescence at 520 nm was reached. The reactants were dissolved in 5 mM KCl, 10 mM MgCl_2_, 10 mM Tris-HCl, pH 8.7. The pseudo first order kinetics, which is equal to *k*_on_ x [BHQ-RC24], was estimated from the exponential decay fitting of the experimental points.

In the off-rate assay, QMC dissociation is accompanied by fluorescence recovery. In solution, containing 0.16 μM FAM-LC23 and 1 μM BHQ-RC24, was added 16 μM RC24 without BHQ attachment and fluorescence measurements were immediately started. Thus, the assay was performed in the presence of excess RC24, so that dissociated FAM-LC23 with recovered fluorescence are coupled with the latter and stays fluorescent.

### Preparation of QMC-coated beads

QMC-beads were prepared according to the manufactures recommendations using 0.5, 1, 2 and 5 nmol amino modified RC24 per 1 mg beads (Dynabeads MyOne carboxylic acid, Invitrogen). Specifically, 200 μL bead solution was washed twice on a magnet in 0.6 mL 100 mM MES, pH 4.8. In a separate tube, needed amount of RC24 was mixed with EDC just before use. RC24/EDC mixture was added to the magnetic beads, mixed by 10 sec vortexing and incubated on a rotating mixer at ambient temperature overnight. The next day, beads were washed three times in 0.6 mL 0.01% Tween-20, 250 mM Tris-HCl, pH 8 (each round included incubation for 30 min) and resuspended in 200 μL 5 mM KCl, 10 mM MgCl_2_, 10 mM Tris pH 8.7 resulting in 10 mg/mL final concentration of the magnetic beads.

### Immobilization of FAM-LC23 to QMC-coated beads

Typically, 60 μL QMC-MB (10 mg/ml) was resuspended in 50 μL 5 mM KCl, 10 mM MgCl_2_, 10 mM Tris pH 8.7. 12 μL (10 μM) FAM-LC23 was added into the suspension, mixed by pipetting, incubated for 15 min at 60 °C (mixing each 5 min). The beads were washed three times in 0.6 mL 5 mM KCl, 10 mM MgCl_2_, 10 mM Tris pH 8.7 buffer (first wash was performed at 60 °C and others at room temperature), resuspended in 300 μL final buffer and stored at 4 °C.

The binding capacity of the QMC-beads was estimated by measuring fluorescence of supernatants before and after decoupling the QMC by EDTA solution (25 mM EDTA, 10 mM Tris-HCl) at 80 °C for 5 min. The thermal stability of QMC was studied similarly. After immobilization of FAM-LC23, 100 μL suspension was kept at desired temperature for 5 min and fluorescence of the supernatants was measured to estimate released FAM-LC23.

### Immobilization of FAM-T20 to streptavidin-coated bead**s**

Streptavidin-covered beads (Dynabeads MyOne Streptavidin C1, Invitrogen) were prepared similarly with the following differences: (i) washing, binding capacity and thermal stability studies were performed in1 M NaCl, 0.5 mM EDTA, 5 mM Tris-HCl, pH 7.5, and (ii) after adding FAM-T_20_-biotin, the suspension was incubated for 15 min at room temperature using gentle rotation.

## Additional Information

**How to cite this article**: Kankia, B. Quadruplex-and-Mg^2+^ Connection (QMC) of DNA. *Sci. Rep.* **5**, 12996; doi: 10.1038/srep12996 (2015).

## Supplementary Material

Supplementary Information
