# Supplementary material for: Quadruplex-and-Mg2+ Connection (QMC) of DNA
Source: Sci Rep. 2015 Aug 12;5:12996. doi: 10.1038/srep12996 (PMC4533012; doi:10.1038/srep12996)
Supplement: Supplementary Information [file srep12996-s1.pdf]

SUPPLEMENTARY INFORMATION

Quadruplex-and-Mg^{2+} Connection (QMC) of DNA

Besik Kankia

Department of Chemistry and Biochemistry, The Ohio State University, Columbus OH 43210, USA

E-mail: kankia.1@osu.edu, Telephone: 614-688-8799, Fax: 614-688-5402

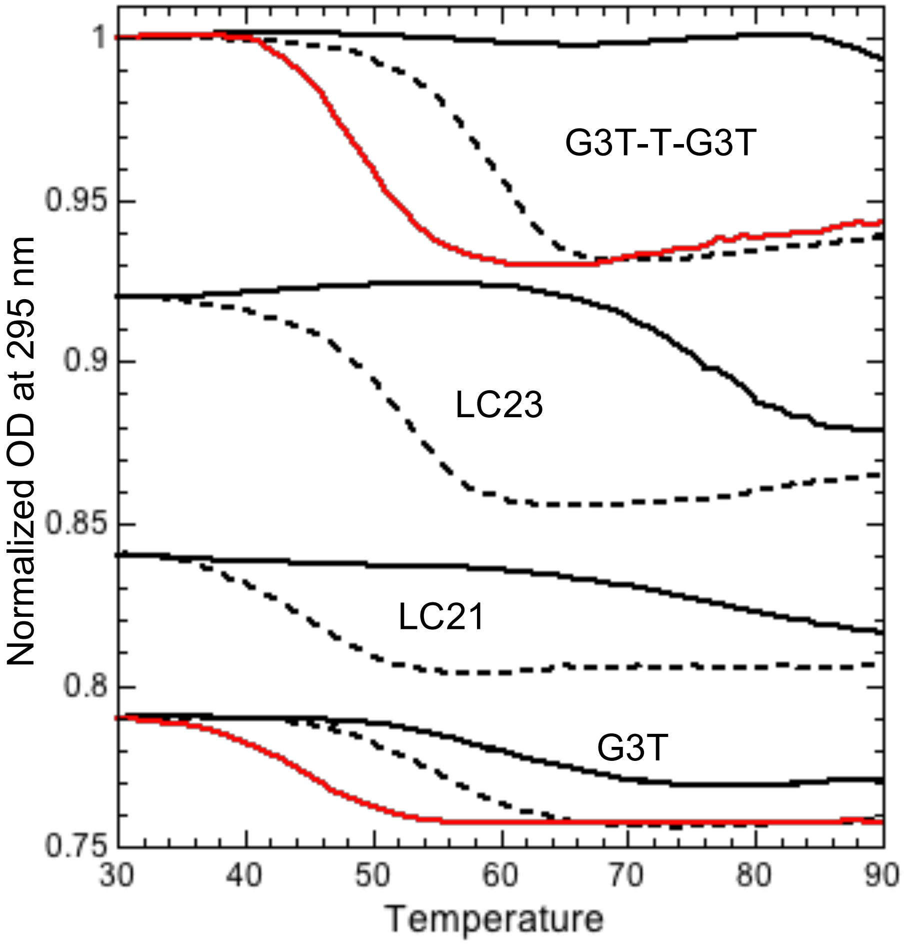

Figure S1. UV meltings of $(G3T)_2$ variants in 0.1 mM K^+ in the absence (dashed lines) and presence of 1 mM Mg^{2+} (black solid lines) and 100 mM Cs^+ (red lines). G3T-T-G3T has thymidine in the middle, which inhibits uninterrupted tetrahelical structure in the absence of Mg^{2+} ions. Adding Mg^{2+} results in more than 40 °C stabilization, which we attribute to recovery of the fold. LC23 and LC21 represent truncated versions, missing G and TGGG at the 3'-end. Both variants reveal 25-30 °C stabilization effect upon adding Mg^{2+} ions. While Mg^{2+} strongly stabilizes $(G3T)_2$ variants, the stabilization effect on the G3T monomer (bottom) is only ~ 5 °C. Cs^+ ions were employed to demonstrate that Mg^{2+} stabilization effect is not just an ionic effect. Cs^+ ions demonstrate even destabilization due to decreasing the effective concentration of K^+ ions. For clarity, the curves corresponding to LC23, LC21 and G3T are offset by 0.08, 0.16 and 0.21 OD units, respectively.

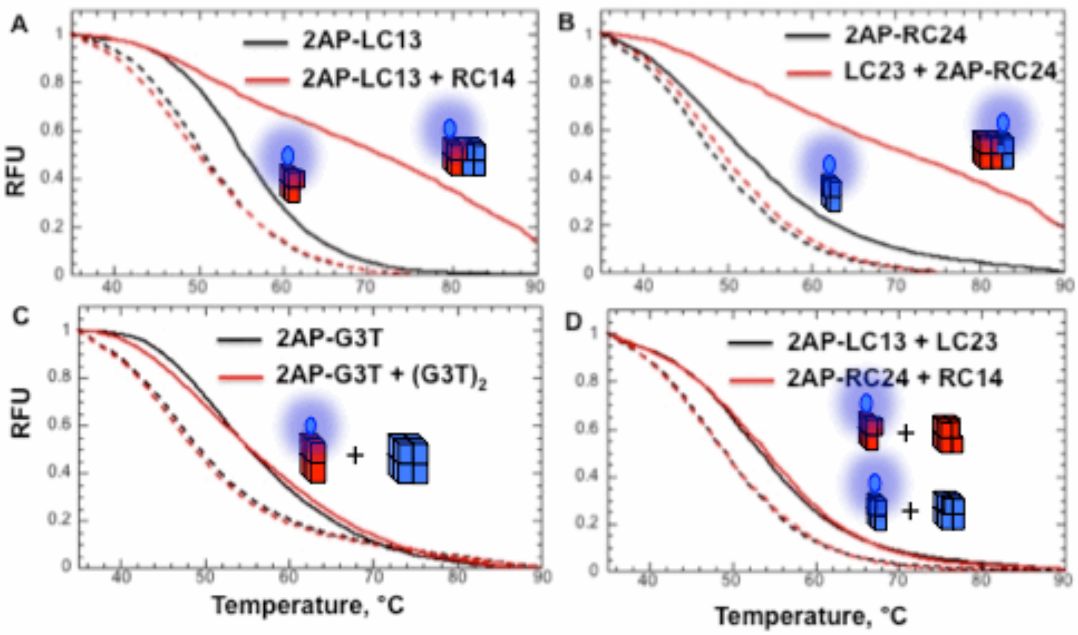

Figure S2. Fluorescence melting experiments in 0.1 mM K^+ in the absence (dashed lines) and presence (solid lines) of 1 mM Mg^{2+}. Panels A, B and C represent the melts of the matching QMC partners LC13/RC14, LC23/RC24 and LC12/RC13, respectively. Panel D demonstrates cross-binding experiment between LC13/RC14 and LC23/RC24. The stabilization effect of Mg^{2+} on the separate connectors, blunt-ended system (panel C) and cross-binding partners (panel D) demonstrates a moderate, ~5 °C, increase in T_m. Only QMC partners with the shape complementarity or locking mechanism reveal strong stabilization (panels A and B) characteristic of QMC formation.

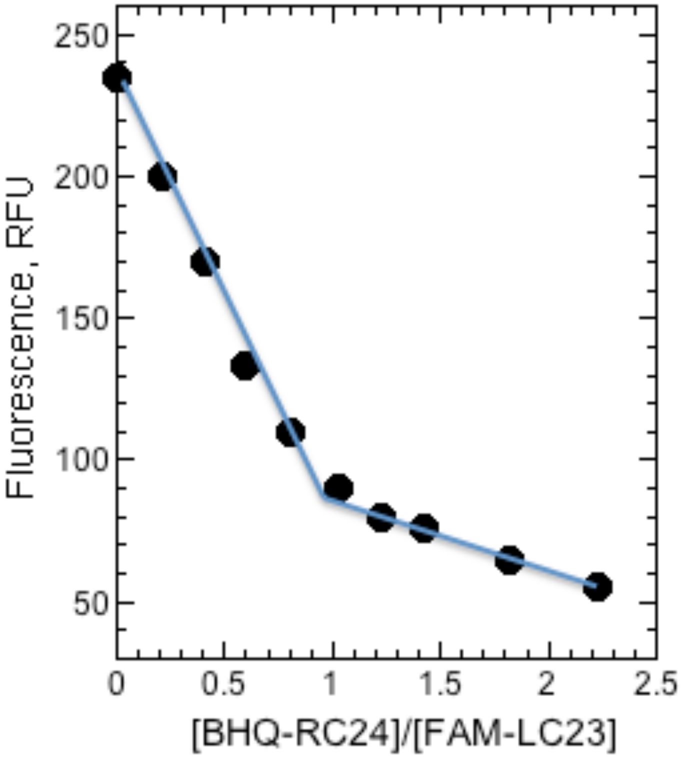

F**igure S3**. Fluorescence titration curves of FAM-LC23 with BHQ-RC24 at 50 °C. Buffer: 5 mM KCl, 2 mM $MgCl_2$, 10 mM Tris-HCL, pH 8.7. Initial concentration fo FAM-LC23 is 250 nM.

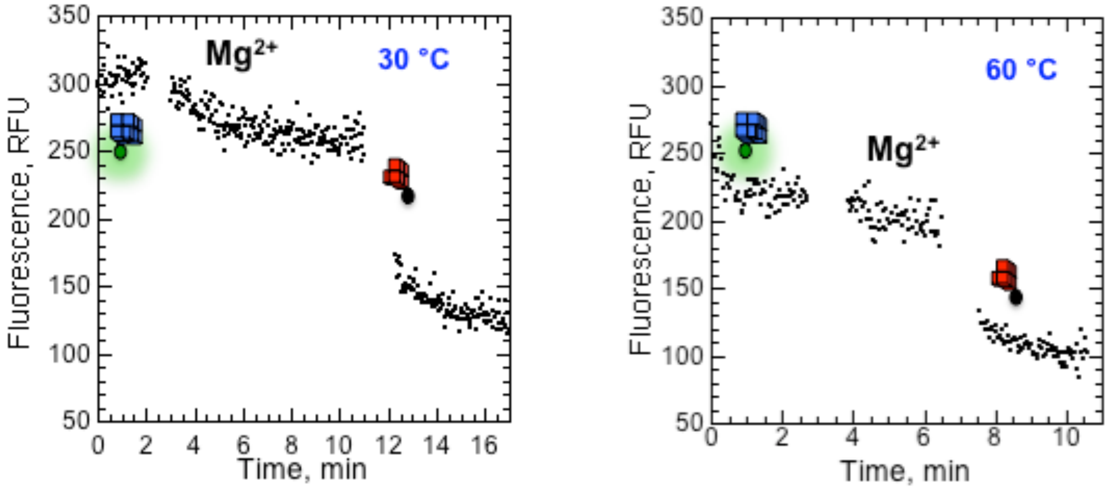

Figure S4. Fluorescence effects of FAM-LC23 upon adding 10 mM $MgCl_2$ and BHQ-RC24. Buffer: 5 mM KCl, 10 mM Tris HCl; [FAM-LC23] = 250 nM, [BHQ-RC24] = 500 nM.

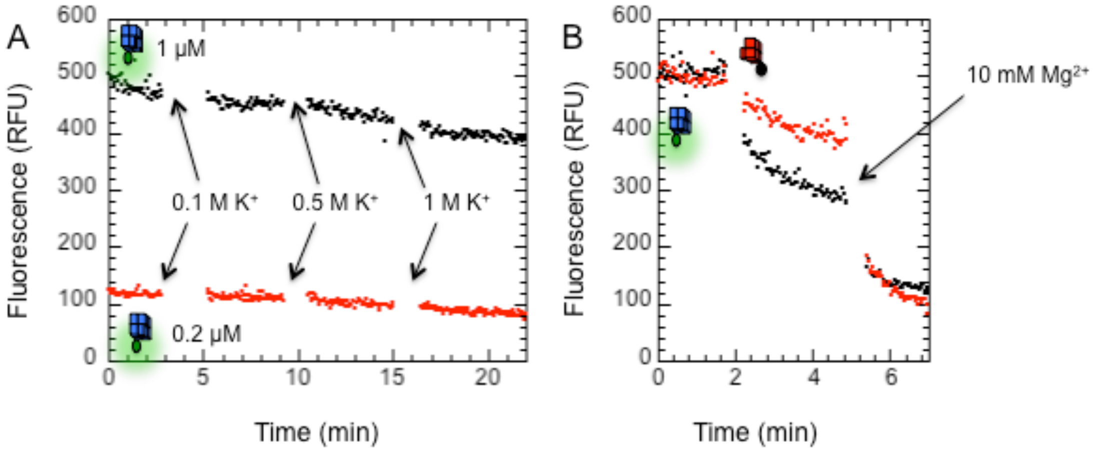

Figure S5. Role of K^+ ions in QMC. (A) Titration of 1 μM (black) and 0.2 μM (red) FAM-LC23 by KCl. Initial concentration of KCl is 1 mM. (B) Quenching of FAM-LC23 in 0.1 M KCl (red) and 1 M KCl (black) upon adding BHQ-RC24. To complete the quenching, 25 mM $MgCl_2$ was added around 5 min. [FAM-LC23] = 1 μM, [BHQ-RC24] = 1.25 μM.

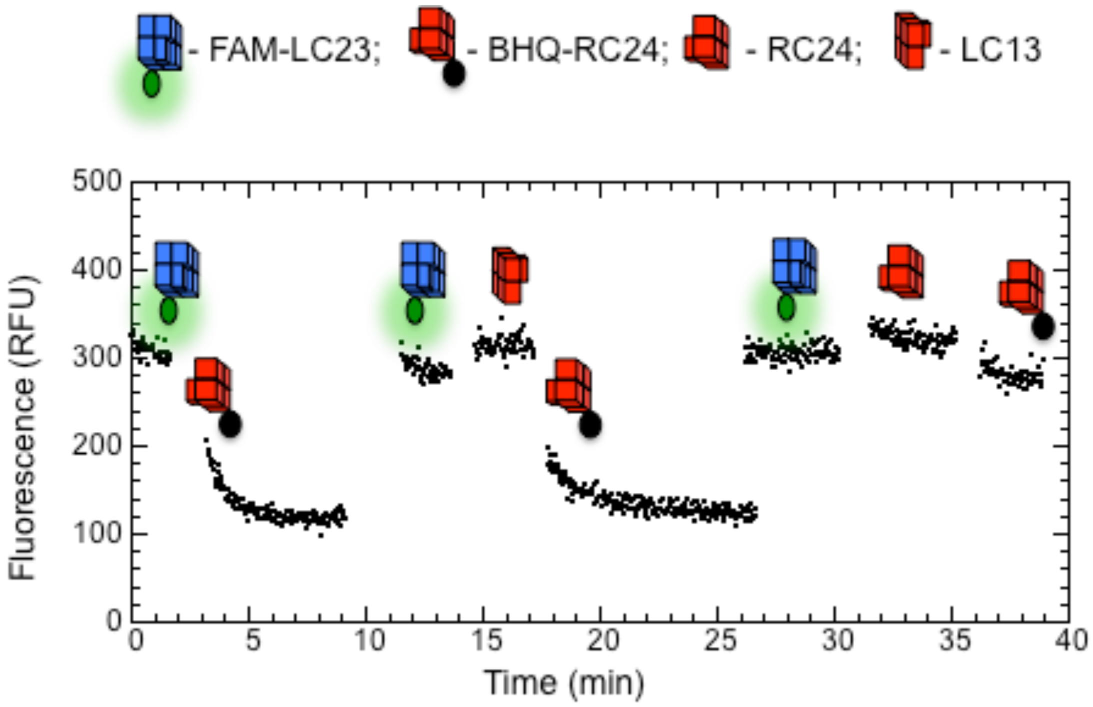

Figure S6. Effect of mismatched and matched binding partners on QMC (LC23/RC24) formation at 60 °C. The graph presents three mixing experiments. All experiments starts with 250 nM FAM-LC23 dissolved in 5 mM KCl and 2 mM $MgCl_2$. Left experiment is the positive control demonstrating rapid fluorescence quenching upon adding matched binding partner with tagged quencher, 500 nM BHQ-LC24. In the middle experiment, before adding BHQ-LC24, mismatched or cross-binding partner from another QMC connection (see Fig. 1), LC13 (2 µM), was added. However, LC13 could not interfere into the QMC formation upon addition of BHQ-LC24. This clearly indicates that the cross-binding partners do not form a stable complex. In the last experiment, matching LC24 without tagged quencher ("cold" version of BHQ-LC24) was used, which completely inhibited QMC formation.

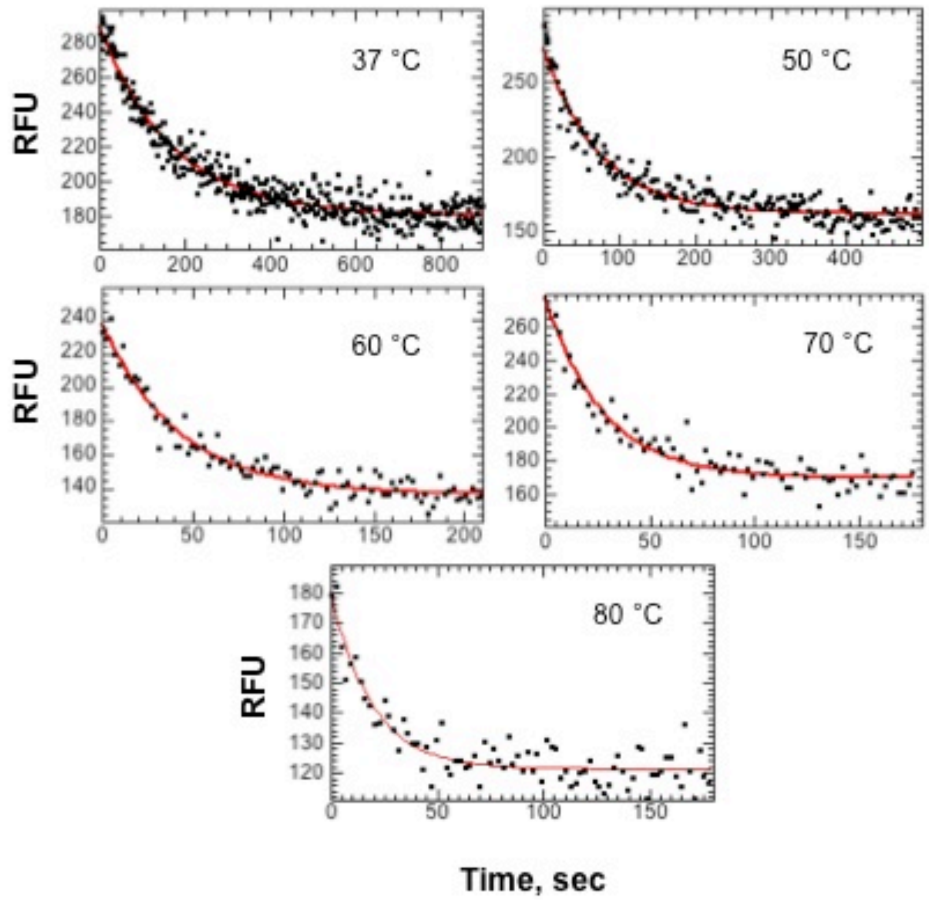

Figure S7. Stopped-flow kinetics of QMC formation at different temperatures initiated by mixing equal volumes of FAM-LC23 and BHQ-RC24 dissolved in 5 mM KCl, 10 mM $MgCl_2$ and 10 mM Tris-HCl**.** The on-rate constants, *k*, were estimated from a single-exponential fit (red) of the experimental data.
